# International society of sports nutrition position stand: diets and body composition

**DOI:** 10.1186/s12970-017-0174-y

**Published:** 2017-06-14

**Authors:** Alan A. Aragon, Brad J. Schoenfeld, Robert Wildman, Susan Kleiner, Trisha VanDusseldorp, Lem Taylor, Conrad P. Earnest, Paul J. Arciero, Colin Wilborn, Douglas S. Kalman, Jeffrey R. Stout, Darryn S. Willoughby, Bill Campbell, Shawn M. Arent, Laurent Bannock, Abbie E. Smith-Ryan, Jose Antonio

**Affiliations:** 1 Department of Family and Consumer Sciences, California State University, Northridge, CA USA; 2 Department of Health Sciences, Lehman College, Bronx, NY USA; 3 Dymatize Nutrition, Dallas, TX USA; 4 High Performance Nutrition, Mercer Island, WA USA; 5 Department of Exercise Science and Sport Management, Kennesaw State University, Kennesaw, GA USA; 6 Department of Exercise and Sports Science, University of Mary Hardin-Baylor, Belton, TX USA; 7 Exercise and Sports Nutrition Laboratory, Texas A&M University, College Station, TX USA; 8 Health and Exercise Science, Skidmore College, Saratoga Springs, NY USA; 9 Nutrition Research Division, QPS, Miami, FL USA; 10 Institute of Exercise Physiology and Wellness, University of Central Florida, Orlando, FL USA; 11 Department of Health, Human Performance and Recreation, Baylor University, Waco, TX USA; 12 Performance & Physique Enhancement Laboratory, Exercise Science Program, University of South Florida, Tampa, FL USA; 13 Department of Kinesiology & Health, IFNH Center for Health & Human Performance, Rutgers University, New Brunswick, NJ USA; 14 Guru Performance Institute, Norwich, UK; 15 Department of Exercise and Sport Science, University of North Carolina, Chapel Hill, NC USA; 16 Department of Health and Human Performance, Nova Southeastern University, Davie, FL USA

## Abstract

Position Statement: The International Society of Sports Nutrition (ISSN) bases the following position stand on a critical analysis of the literature regarding the effects of diet types (macronutrient composition; eating styles) and their influence on body composition. The ISSN has concluded the following. 1) There is a multitude of diet types and eating styles, whereby numerous subtypes fall under each major dietary archetype. 2) All body composition assessment methods have strengths and limitations. 3) Diets primarily focused on fat loss are driven by a sustained caloric deficit. The higher the baseline body fat level, the more aggressively the caloric deficit may be imposed. Slower rates of weight loss can better preserve lean mass (LM) in leaner subjects. 4) Diets focused primarily on accruing LM are driven by a sustained caloric surplus to facilitate anabolic processes and support increasing resistance-training demands. The composition and magnitude of the surplus, as well as training status of the subjects can influence the nature of the gains. 5) A wide range of dietary approaches (low-fat to low-carbohydrate/ketogenic, and all points between) can be similarly effective for improving body composition. 6) Increasing dietary protein to levels significantly beyond current recommendations for athletic populations may result in improved body composition. Higher protein intakes (2.3–3.1 g/kg FFM) may be required to maximize muscle retention in lean, resistance-trained subjects under hypocaloric conditions. Emerging research on very high protein intakes (>3 g/kg) has demonstrated that the known thermic, satiating, and LM-preserving effects of dietary protein might be amplified in resistance-training subjects. 7) The collective body of intermittent caloric restriction research demonstrates no significant advantage over daily caloric restriction for improving body composition. 8) The long-term success of a diet depends upon compliance and suppression or circumvention of mitigating factors such as adaptive thermogenesis. 9) There is a paucity of research on women and older populations, as well as a wide range of untapped permutations of feeding frequency and macronutrient distribution at various energetic balances combined with training. Behavioral and lifestyle modification strategies are still poorly researched areas of weight management.

## Background

There are several major diet types interspersed with a multitude of subtypes. This creates a maze of conflicting principles that may be difficult for the general public and practitioners to navigate. Compounding the confusion is the continued propagation of fad diets across a range of media outlets, replete with unfounded practices. Therefore, it is important to examine the scientific evidence in a systematic way in order to devise recommendations to guide healthcare practitioners, coaches (including trainers, dietitians, and sports nutritionists), athletes, and the general public regarding all of the above. The purpose of this position stand is to provide clarity on the effects of various diets on body composition.

A general definition of “diet” is the sum of energy and nutrients obtained from foods and beverages consumed regularly by individuals. Thus, the following dietary archetypes will be assessed: very-low- and low-energy diets (VLED and LED), low-fat diets (LFD), low-carbohydrate diets (LCD), ketogenic diets (KD), high-protein diets (HPD), and intermittent fasting (IF). Diets with qualitative themes or commercial brands will inevitably fall under the umbrella of the classifications above. Therefore, their parent categories rather than ‘named’ or ‘branded’ diets (e.g., Atkins, Ornish, Zone, Paleo, etc.) will receive the majority of scrutiny in this position stand.

This position stand will further focus on prospective intervention trials with a duration of at least 4 weeks, as this can be considered a minimum period of time for meaningful changes in fat mass (FM) and lean mass (LM, termed interchangeably with fat-free mass, or FFM), as well as effects of exercise training on these variables. Studies and pooled analyses with and without training have been included, as well as studies across the range of energetic balances (i.e., hypo-, hyper-, and eucaloric). Studies that did not measure body composition have not been included, nor have studies examining dietary effects in clinical contexts – including disease treatment. Despite the latter topics breaching the scope of the present article, it is still important to note that body composition is inextricably tied to foundational parameters of health. Aside from sports and fitness applications for improvements in body composition, a higher proportion of LM reduces the risk of developing metabolic syndrome [1], bone loss [2], and the multiple complications associated with sarcopenia [3, 4].

## Body composition assessment methods

Body composition assessment is an attempt to simplify a process that is inherently complex. As such, there are several methods that attempt to accurately estimate LM and FM, and their subcomponents. Before outlining the most common methods used in sports science and medicine, it should be noted that there is a continuum of the components measured or estimated. Over 25 years ago, Wang et al. [5] proposed a five-level model for organizing body composition research [6]. Each level has different components, eventually deemed compartments, and have undergone further organization to include two (2C), three (3C) and four (4C) compartments [6]:

1. *Atomic level:* hydrogen, oxygen, nitrogen, carbon, sodium, potassium, chloride, phosphorus, calcium, magnesium, sulfur.
2. *Molecular level:* The 4C model includes FM, total body water (TBW), total body protein, and bone mineral content. The 3C model includes FM, TBW, and nonfat solids. An alternate 3C model includes FM, bone mineral, and residual mass. The 2C model includes FM and FFM.
3. *Cellular level:* The 3C model includes cells, extracellular fluids, and extracellular solids. The 4C model includes body cell mass, FM, extracellular fluids, and extracellular solids.
4. *Tissue-organ level:* adipose tissue, skeletal muscle, bone, visceral organs, other tissues.
5. *Whole body level:* head, trunk, and appendages.

The 4C model has the greatest degree of sensitivity to interindividual variability of FFM composition. Its comprehensiveness and accuracy have rendered its reputation as the “gold standard” to which all other models are compared, but it is limited to occasional use in primary research due to its logistical challenges. The 2C model estimates FM and FFM, and operates under the assumption that water, protein, and mineral content of FFM are constant. Thus, the 2C model is the most commonly used approach for adults. Due to their relatively low cost, non-invasiveness, and ease of operation, 2C model-based methods are common in clinical practice and sports/fitness settings. Examples of methods based on the 2C model include hydrodensitometry (underwater weighing), air displacement plethysmography (ADP or BOD POD**®**), skinfold thickness, and bioelectrical impedance analysis (BIA). Dual energy X-ray absorptiometry (DXA) is based on a 3C model that measures bone mineral content, LM, and FM, but it is still subject to confounding from inter-assessment differences in hydration, glycogen, and muscle creatine levels, which can be significant in athletic populations with distinct exercise and recovery cycles [7, 8].

Body composition methods have been further classified as direct, indirect, and criterion [9]. Direct methods measure the specific/targeted aspect or process. Examples include TBW, isotope dilution, and neutron activation. Indirect methods provide surrogate measures or proxies of direct methods and criterion methods. Examples of indirect methods are anthropometry (e.g., skinfolds), ADP, BIA, and bioimpedance spectroscopy (BIS). Criterion methods measure a specific property of the body such as density or distribution of skeletal muscle and adipose tissue. Examples include hydrodensitometry, computed tomography, magnetic resonance imaging (MRI), and DXA. It should be noted that multi-compartment models have evolved to be considered criterion methods: standards against which other methods are judged.

The various methods are often classified in the literature as either laboratory methods (e.g., DXA, ADP) or field methods (e.g., skinfolds, ultrasound, BIA, BIS) depending on their respective use in research and clinical settings as well as their portability. Laboratory methods – including multi-compartment models – have traditionally been viewed as more accurate and valid. BIA and BIS have evolved to include multiple frequencies. This technology may more accurately estimate body composition through multiple frequency-dependent electrical properties of body tissues, as opposed to traditional single frequency methods (i.e., handheld BIA or scales). However, higher levels of sophistication with multi-frequency options are often accompanied by lower availability and higher cost. Given the broad range of body composition measurement techniques and unique challenges involved with measuring athletes (exercise/glycogen depletion, hydration, time availability, etc.), there is no universally superior method for body composition assessment in this population [10–12]. An excellent review by Wagner and Hayward [10] concludes the following: “There is no single method which is ‘best;’ rather, the clinician or researcher must weigh the practical considerations of their assessment needs with the limitations of the methods.” Table 1 outlines the characteristics of selected body composition assessment methods [6, 9, 10, 13–20]:

**Table 1:** Body composition methods

Method	Components measured/estimated	Strengths	Limitations
Skinfold thickness	Subcutaneous fat thickness in specific sites of the body	Reliable method for assessing regional fatness. Useful for monitoring fat changes in children due to their small body size, and their fat stores are primarily subcutaneous, even in obese children (though increasing degrees of obesity lower the viability of this method).	Most skinfold calipers have an upper limit of 45–60 mm, limiting their use to moderately overweight or thin subjects. Measurement reliability depends on the skill and experience level of the technician, which varies, and type/brand of caliper used. The best use of this method is the monitoring of raw values, rather than assuming an accurate representation of body composition.
Bioelectrical impedance analysis (BIA) and bioelectrical impedance spectroscopy (BIS)	Total body water (TBW), which is converted to FFM via the assumption that 73% of the body’s FFM is water	Economical, safe, quick, minimal participant participation and technician expertise. Capable of determining body composition of groups and monitoring changes within individuals over time. BIS or multi-frequency BIA, is capable of delineating TBW into intracellular water (ICW) and extracellular water (ECW), which allows for an estimation of body cell mass.	Validity of BIA and BIS is population-specific; it’s influenced by sex, age, height, disease state, and race. BIA/BIS underestimates FFM in normal-weight individuals and overestimates FFM in obese individuals compared to DXA. Validity of single-frequency BIA and multifrequency BIA may be limited to healthy, young, euhydrated adults.
Hydrodensitometry (also called hydrostatic weighing or underwater weighing)	Body weight on land and weight in water, body volume, body density, and residual lung volume	Good test-retest reliability, accurate in determining body density, lengthy history and track record of consistent use in sports and clinical settings.	Relies upon subject performance (completely exhaled, submerged). Errors in measurement of residual lung volume can confound the assessment of body composition. The density of FFM is an assumed constant but can vary with age, sex, race, and training status.
Air displacement plethysmography (ADP)	Total body volume, and total body fat (FFM and FM)	High reliability for body fat percentage, body density, and residual lung volume in adults. Non-invasive, quick, no radiation exposure or subject performance demands. Same-day test-retest reliability has been reported to be slightly better than hydrodensitometry	Tends to over-estimate fat mass compared to DXA and the 4C model. Disease states can reduce accuracy. Inconsistency of clothing and facial/body hair and exercise prior to testing can alter repeatability. Expensive apparatus.
Dual energy X-ray absorptiometry (DXA)	Total and regional body fat, LM, bone mineral density	High accuracy and reproducibility for all age groups. Non-invasive, quick, no subject performance needed. Measurements are not confounded by disease states or growth disorders. Gold standard for diagnosing osteopenia and osteoporosis.	Small amount of radiation exposure. Fat mass estimates are confounded by trunk thickness (error increases alongside degree of trunk thickness). Compared to 4C, DXA may be unreliable for longitudinal studies of subjects who undergo major changes in glycogen or hydration status between measurements. Expensive apparatus.
Ultrasound	Tissue layer thickness (skin, adipose, muscle)	Highly repeatable, readily available, widely used, portable, quick. Noninvasive and no ionizing radiation. Accurate and precise estimates of fat thickness in multiple sites of the body, capable of measuring the thickness of muscle and bone.	Requires a skilled, experienced technician. Measurement procedures and techniques are not yet standardized. Inherent confounders such as fascia can complicate the interpretation of results. Higher cost than field methods.
Magnetic resonance imaging (MRI) and computed tomography	Total and regional fat (including subcutaneous and visceral), skeletal muscle, organs and other internal tissues, lipid content in muscle and liver	High accuracy and reproducibility. MRI does not involve exposure to radiation.	Expensive, lengthy procedure. Limited to accommodating normal to moderately overweight individuals, but not very large body sizes do not fit in the field of view. High radiation exposure with computed tomography.
Near-infrared interactance (NIR)	Fat, protein, and water – based on assumptions of optical density	Good test-retest and day-to-day reliability. Quick, non-invasive.	Large standard errors of estimation (SEE > 3.5% BF). Percent body fat is systematically underestimated, and this error increases alongside larger body frames.

## Major diet archetypes

### Low-energy diets

Low-energy diets (LED) and very-low-energy diets (VLED) are characterized by their provision of 800–1200 kcal/day and 400–800 kcal/day, respectively [21]. Note that LED have also been given a more liberal definition of providing 800–1800 kcal [22]. Very-low-energy diets are typically in liquid form and commercially prepared. The aim of the diet is to induce rapid weight loss (1.0–2.5 kg/week) while preserving as much LM as possible. VLED are designed to replace all regular food consumption, and therefore should not be confused with meal replacement products intended to replace one or two meals per day. As such, VLED are fortified with the full spectrum of essential micronutrients. The macronutrient content of VLED is approximately 70–100 g/day, 15 g/day and 30–80 g/day of protein, fat and carbohydrate, respectively. A protein-sparing modified fast can be considered the higher-protein variant of a VLED, with protein intakes of approximately 1.2–1.5 g/kg/d [23]. However, even at protein intakes as low as 50 g/day, the proportion of LM loss from VLED has been reported to be 25% of total weight loss, with 75% as fat loss [24].

Resistance training has shown an impressive ability to augment the preservation of muscle and even increase it during VLED – at least in untrained/obese subjects. A 12-week trial by Bryner et al. [25] found that resistance training while consuming 800 kcal resulted in the preservation of LM in untrained obese subjects. There was actually a slight gain, but it did not reach statistical significance. Resting metabolic rate (RMR) significantly increased in the training group, but it decreased in the control group. Donnelly et al. [26] reported a significant increase in cross-sectional area of both slow- and fast-twitch muscle fibers in untrained obese subjects after 12 weeks on an 800 kcal diet with resistance training. While these results cannot necessarily be extrapolated to lean, trained subjects, they are nevertheless intriguing.

In obese populations, aggressive caloric restriction is a potentially powerful intervention since a greater initial weight loss is associated with greater long-term success in weight loss maintenance [27]. However, a meta-analysis by Tsai and Wadden [22] found that VLED did not result in greater long-term (1 year or more) weight loss than LED. Eight to 12 week VLED are common in clinical practice before transitioning to less severe caloric restriction; however, there is an ongoing debate regarding the duration that can be safely sustained for VLED. Multiple deaths have been reported due to low-quality protein intake, excessive loss of lean mass, and inadequate medical supervision [28]. Adverse effects of VLED include cold intolerance, fatigue, headache, dizziness, muscle cramps, and constipation. Hair loss was reported to be the most common complaint of extended VLED use [22]. It should be noted that VLED use has limited relevance to healthy and athletic populations.

### Low-Fat diets

Low-fat diets (LFD) have been defined as providing 20–35% fat [29]. This is based on the Acceptable Macronutrient Distribution Ranges (AMDR) for adults, set by the Food and Nutrition Board of the Institute of Medicine [30]. The AMDR set protein at 10–35%, carbohydrate at 45–65%, and fat at 20–35% of total energy. Although the classification of LFD is based on the AMDR, it might be more accurate to call them high-carbohydrate diets, given the dominance of this macronutrient in the ranges. As such, the definition of LFD is inherently subjective.

Scientists and physicians have promoted decreased fat intake since the 1950s [31]. The 1977 publication of the *Dietary Goals for the United States*, and the 1980 publication of the inaugural *Dietary Guidelines for Americans* (DGA) reinforced a reduction in total fat intake with the aim of improving public health [32]. Although the AMDR were published in 2005, their staying power is apparent since the recently updated DGA adheres to these ranges [33], as do major health organizations such as the American Heart Association, American Diabetes Association and Academy of Nutrition and Dietetics.

A recent systematic review by Hooper et al. [34] analyzed 32 randomized controlled trials (RCTs) containing ~54,000 subjects, with a minimum duration of 6 months. Reducing the proportion of dietary fat compared to usual intake modestly but consistently reduced body weight, body fat, and waist circumference. Excluded from the analysis were RCTs where subjects in either the control or experimental groups had the intention to reduce weight. The implication of these findings is that reducing the proportion of dietary fat can cause a de facto reduction of total energy intake, thereby reducing body fat over time.

The premise of dietary fat reduction for weight loss is to target the most energy-dense macronutrient to impose hypocaloric conditions. Tightly controlled experiments have covertly manipulated the fat content of diets similar in appearance and palatability, and the higher energy density of the higher-fat diets resulted in greater weight gain and/or less weight loss [35, 36]. However, over the long-term, diets with lower energy density have not consistently yielded greater weight loss than energy restriction alone [37, 38]. Reasons for the disparity between short- and long-term effects of energy density reduction include speculation that learned compensation is occurring. In addition, postprandial factors may increase sensory-specific satiety that over time can reduce the initial palatability of energy-dense foods [39].

Very-low-fat diets (VLFD) have been defined as providing 10–20% fat [29]. Diets fitting this profile have a limited amount of research. The body of controlled intervention data on VLFD mainly consists of trials examining the health effects of vegetarian and vegan diets that aggressively minimize fat intake. These diets have shown consistently positive effects on weight loss [40], but this literature lacks body composition data. Among the few studies that did, the A TO Z Weight Loss Study by Gardner et al. [41], did not show any significant between-group differences in body fat reduction among the diets (Atkins, Zone, LEARN, and Ornish). However, despite the Ornish group’s assigned fat intake of ≤10% of total calories, actual intake progressed from 21.1 to 29.8% by the end of the 12-month trial. Similar results were seen by de Souza et al. [42] in the POUNDS LOST trial. Four groups were assigned high-protein (25%) and average-protein (15%) versions of high-fat (40%) and low-fat (20%) diets. No significant between-group differences were seen in the loss of total abdominal, subcutaneous, or visceral fat at either six months or two years. A mean loss of 2.1 kg LM and 4.2 kg FM occurred in both groups at 6 months. No LM-retentive advantage was seen in the higher-protein diets, but this could have been due to both protein intake levels being sub-optimal (1.1 and 0.7 g/kg). As seen in previous LFD research, the targeted restriction to 20% fat was apparently difficult to attain since actual intakes ranged 26–28%.

### Low-carbohydrate diets

Similar to LFD, low-carbohydrate diets (LCD) are a broad category lacking an objective definition. There is no universal agreement on what quantitatively characterizes an LCD. The AMDR lists 45–65% of total energy as the appropriate carbohydrate intake for adults [33]. Therefore, diets with intakes below 45% fall short of the ‘official’ guidelines and can be viewed as LCD. However, other published definitions of LCD disregard the limits set in the AMDR. LCD have been defined as having an upper limit of 40% of total energy from carbohydrate [43, 44]. In absolute rather than proportional terms, LCD have been defined as having less than 200 g of carbohydrate [43]. Some investigators have taken issue with this liberal definition of LCD, preferring to delineate non-ketogenic LCD as containing 50–150 g, and KD as having a maximum of 50 g [45].

Meta-analyses comparing the effects of LFD with LCD have yielded mixed results across a wide range of parameters. Liberal operational definitions of LCD (e.g., ≤45%) have led to a lack of significant differences in body weight and waist circumference [46], while lower carbohydrate classification thresholds (<20%) have favored LCD for weight loss and other cardiovascular risk factors [47]. Recently, Hashimoto et al. [48] conducted the first-ever meta-analysis on the effect of LCD on fat mass (FM) and body weight. The analysis, limited to trials involving overweight/obese subjects, had a total of 1416 subjects, stratifying the diets as “mild LCD” (~40% CHO) or “very LCD” (~50 g CHO or 10% of total energy). Eight RCTs included a very LCD treatment, and 7 RCTs included a mild LCD treatment. With all groups considered, FM decrease was significantly greater in the LCD than the control diets. However, sub-analysis showed that fat mass decrease in very LCD was greater than the controls, while the difference in FM decrease between mild LCD and controls was not significant. A separate sub-analysis of short- versus long-term effects found that both types of LCD yielded significantly greater fat loss than controls in trials less than, as well as longer than 12 months. A further sub-analysis of found that BIA failed to detect significant between-group differences in FM reduction, while DXA showed significantly greater decreases in LCD than controls. It should be noted that despite reaching statistical significance, mean differences in FM reduction between LCD and control groups were small (range = 0.57–1.46 kg). Practical relevance is questionable given the obese nature of the subjects. The authors speculated that the advantage of the LCD over the control diets could have been due to their higher protein content.

### Ketogenic diets

Despite being a subtype of LCD, the ketogenic diet (KD) deserves a separate discussion. Whereas non-ketogenic LCD is subjectively defined, KD is objectively defined by its ability to elevate circulating ketone bodies measurably – a state called ketosis, also known as physiological or nutritional ketosis. Aside from completely fasting, this condition is attained by restricting carbohydrate to a maximum of ~50 g or ~10% of total energy [45], while keeping protein moderate (1.2–1.5 g/kg/d) [49], with the remaining predominance of energy intake from fat (~60–80% or more, depending on degree protein and carbohydrate displacement). Ketosis is a relatively benign state not to be confused with ketoacidosis, which is a pathological state seen in type 1 diabetics, where a dangerous overproduction of ketones occurs in the absence of exogenous insulin. The primary ketone produced hepatically is acetoacetate, and the primary circulating ketone is β-hydroxybutyrate [50]. Under normal, non-dieting conditions, circulating ketone levels are low (<3 mmol/l). Depending on the degree of restriction of carbohydrate or total energy, KD can raise circulating ketone levels to a range of ~0.5–3 mmol/l, with physiological ketosis levels reaching a maximum of 7–8 mmol/l [49].

The proposed fat loss advantage of carbohydrate reduction beyond a mere reduction in total energy is based largely on insulin-mediated inhibition of lipolysis and presumably enhanced fat oxidation. However, a single-arm study by Hall et al. [51] examined the effect of 4 weeks on a low fat diet (300 g CHO) followed by 4 weeks on a KD (31 g CHO). Blood ketone levels plateaued at ~1.5 mmol/l within two weeks into the KD. A transient increase in energy expenditure (~100 kcal/day) lasting a little over a week occurred upon switching to the KD. This was accompanied by a transient increase in nitrogen loss, potentially suggesting a stress response including the ramping up of gluconeogenesis. Although insulin levels dropped rapidly and substantially during the KD (consisting of 80% fat, 5% CHO), an actual slowing of body fat loss was seen during the first half of the KD phase.

It has been postulated that the production and utilization of ketone bodies impart a unique metabolic state that, in theory, should outperform non-ketogenic conditions for the goal of fat loss [45]. However, this claim is largely based on research involving higher protein intakes in the LCD/KD groups. Even small differences in protein can result in significant advantages to the higher intake. A meta-analysis by Clifton et al. [52] found that a 5% or greater protein intake difference between diets at 12 months was associated with a threefold greater effect size for fat loss. Soenen et al. [53] systematically demonstrated that the higher protein content of low-carbohydrate diets, rather than their lower CHO content, was the crucial factor in promoting greater weight loss during controlled hypocaloric conditions. This is not too surprising, considering that protein is known to be the most satiating macronutrient [54]. A prime example of protein’s satiating effect is a study by Weigle et al. [55] showing that in ad libitum conditions, increasing protein intake from 15 to 30% of total energy resulted in a spontaneous drop in energy intake by 441 kcal/day. This led to a body weight decrease of 4.9 kg in 12 weeks.

With scant exception [56], all controlled interventions to date that matched protein and energy intake between KD and non-KD conditions have failed to show a fat loss advantage of the KD [51, 53, 57–60]. A recent review by Hall [61] states, “There has never been an inpatient controlled feeding study testing the effects of isocaloric diets with equal protein that has reported significantly increased energy expenditure or greater loss of body fat with lower carbohydrate diets.” In light of this and the previously discussed research, the ‘special effects’ of LCD and KD are not due to their alleged metabolic advantage, but their higher protein content. Perhaps the strongest evidence against the alleged metabolic advantage of carbohydrate restriction is a recent pair of meta-analyses by Hall and Guo [60], which included only isocaloric, protein-matched controlled feeding studies where all food intake was provided to the subjects (as opposed to self-selected and self-reported intake). A total of 32 studies were included in the analysis. Carbohydrate ranged from 1 to 83% and dietary fat ranged from 4 to 84% of total energy. No thermic or fat loss advantage was seen in the lower-CHO conditions. In fact, the opposite was revealed. Both energy expenditure (EE) and fat loss were slightly greater in the higher-CHO/lower-fat conditions (EE by 26 kcal/day, fat loss by 16 g/d); however, the authors conceded that these differences were too small to be considered practically meaningful.

A common criticism of the existing literature is that trials need to run longer (several months instead of several weeks) to allow sufficient “ketoadaptation,” which is a physiological shift toward increased fat oxidation and decreased glycogen utilization [62]. The problem with this claim is that the rise in fat oxidation – objectively measured via decreased respiratory quotient – reaches a plateau within the first week of a KD [51]. Increased oxidation of free fatty acids, plasma triacylglycerol, and intramuscular triacylglycerol during exercise is a well-established response to fat-rich diets [63]. However, this rise in fat oxidation is often misconstrued as a greater rate of *net* FM reduction. This assumption ignores the concomitant increase in fat intake and storage. As a result of fat-adaptation, increased intramuscular triacylglycerol levels indicate increased fat synthesis over degradation during the rest periods between exercise bouts [64]. To reiterate a previous point, rigorously controlled isocaloric, protein-matched studies have consistently demonstrated that ketoadaptation does not necessarily amount to a net decrease in fat balance, which is ultimately what matters.

If there is any advantage to KD over non-KD for fat loss, it is potentially in the realm of appetite regulation. Under non-calorically restricted conditions, KD has consistently resulted in body fat and/or body weight reduction [65–69]. This occurs via spontaneous energy intake reduction, which could be due to increased satiety through a suppression of ghrelin production [70]. Moreover, KD has demonstrated hunger-suppressive effects independent of protein content. In a 4-week crossover design, Johnstone et al. [66] found that a KD consumed ad libitum (without purposeful caloric restriction) resulted in an energy intake reduction of 294 kcal/day. The latter results were seen despite a relatively high protein intake (30% of energy) matched between KD (4% CHO) and non-KD (35% CHO) conditions. In further support of this idea, a meta-analysis by Gibson et al. [71] found that KD suppresses appetite more than VLED. However, it remains unclear whether the appetite suppression is due to ketosis or other factors such as an increased protein or fat intake, or restriction of carbohydrate.

An area of growing interest is the effect of KD on athletic performance. Since training capacity has the potential to affect body composition, the effect of KD on exercise performance warrants discussion. Carbohydrate restriction combined with high fat intake to become fat-adapted (or ketoadapted) is a tactic that attempts to improve performance by increasing the body’s reliance on fat as fuel, thereby sparing/decreasing glycogen use, which ostensibly could improve athletic performance. However, in contrast to the proposed benefits of fat-adaptation on performance, Havemann et al. [72] found that 7 days of a high-fat diet (68%) followed by 1 day of high-CHO diet (90%) expectedly increased fat oxidation, but decreased 1-km sprint power output in well-trained cyclists. Stellingwerff et al. [73] compared substrate utilization, glycogenolysis, and enzymatic activity from either 5 days of a high-fat diet (67%) or high-CHO (70%) followed by one day of high-CHO with no training, followed by experimental trials on the seventh day. The high-fat diet increased fat oxidation, but also lowered pyruvate dehydrogenase activity and decreased glycogenolysis. These results provide a mechanistic explanation for the impairment in high-intensity work output as a result of high-fat, CHO-restricted diets [62, 65, 67]. Recently, an ergolytic effect from ketoadaptation has been observed at lower intensities as well. Burke et al. [74] reported that after 3 weeks on a KD at a slight energy deficit, elite race walkers showed increased fat oxidation and aerobic capacity. However, this was accompanied by a reduction in exercise economy (increased oxygen demand for a given speed). The linear and non-linear high-CHO diets in the comparison both caused significant performance improvements, while no significant improvement was seen in the KD (there was a nonsignificant performance decrease). It is notable that Paoli et al. [75] found no decrease in bodyweight-based strength performance in elite artistic gymnasts during 30 days of KD. Furthermore, the KD resulted in significant loss of FM (1.9 kg) and non-significant gain of LM (0.3 kg). However, unlike Burke et al.’s study, which equated protein between groups (~2.2 g/kg), Paoli et al.’s protein intakes were skewed in favor of the KD (2.9 vs. 1.2 g/kg). Wilson et al. [56] recently reported similar increases in strength and power in a protein and calorie-matched comparison of a KD and a Western diet model, suggesting that KD might have less ergolytic potential for strength training than it does for endurance training.

### High-protein diets

A common thread among high-protein diets (HPD) is that they have their various and subjective definitions. High-protein diets have been more generally defined as intakes reaching [76] or exceeding 25% of total energy [29]. High-protein diets have also been identified as ranging from 1.2–1.6 g/kg [54]. Classic work by Lemon et al. showed that protein consumed at double the RDA (1.6 g/kg) repeatedly outperformed the RDA (0.8 g/kg) for preserving LM and reducing FM [77, 78]. However, Pasiakos et al. [79] found that triple the RDA (2.4 g/kg) did not preserve lean mass to a significantly greater extent than double the RDA. More recently, Longland et al. [80] found that in dieting conditions involving high-intensity interval sprints and resistance training, protein intake at 2.4 g/kg caused LM gains (1.2 kg) and fat loss (4.8 kg), while 1.2 g/kg resulted in preservation of lean mass (0.1 kg), and less fat loss (3.5 kg). A unique methodological strength in Longland et al.’s design was the use of the 4C model to assess body composition. Subjects were also provided all food and beverage intake, which added an extra layer of control and strengthened the findings. Augmenting this body of literature is Arciero et al.’s work on “protein-pacing” (4–6 meals/day, >30% protein per meal resulting in >1.4 g/kg/d), which has demonstrated this method’s superiority over conventional, lower-protein/lower-frequency diets for improving body composition in hypocaloric conditions [81, 82].

Of the macronutrients, protein has the highest thermic effect and is the most metabolically expensive. Given this, it is not surprising that higher protein intakes have been seen to preserve resting energy expenditure while dieting [54]. Also, protein is the most satiating macronutrient, followed by carbohydrate, and fat being the least [83]. With just one exception [84], a succession of recent meta-analyses [52, 85–87] supports the benefit of higher protein intakes for reducing body weight, FM, and waist circumference, and preserving LM in an energy deficit. A systematic review by Helms et al. [88] suggested that protein intakes of 2.3–3.1 g/kg FFM was appropriate for lean, resistance trained athletes in hypocaloric conditions. This is one of the rare pieces of literature that report protein requirements on the basis of FFM rather than total body weight.

Antonio et al. [89–92] recently began a series of investigations of which can be considered *super*-HPD. First in the series, the addition of dietary protein amounting to 4.4 g/kg for eight weeks in resistance-trained subjects did not significantly change body composition compared to control conditions of maintenance intake with habitual protein at 1.8 g/kg. Remarkably, the additional protein amounted to an ~800 kcal/day increase, and did not result in additional weight gain. A subsequent 8-week investigation involved resistance-trained subjects on a formally administered, periodized resistance training protocol [90]. The high-protein group (HP) consumed 3.4 g/kg, while the normal-protein group (NP) consumed 2.3 g/kg. HP and NP showed significant gains in LM (1.5 kg in both groups). A significantly greater fat mass decrease occurred in HP compared to NP (1.6 and 0.3 kg, respectively). This is intriguing, since HP reported a significant increase caloric intake compared to baseline (374 kcal), while NP’s caloric increase was not statistically significant (103 kcal). A subsequent 8-week crossover trial [91] in resistance-trained subjects compared protein intakes of 3.3 versus 2.6 g/kg/d. A lack of significant differences in body composition and strength performance were seen despite a significantly higher caloric intake in HP vs. NP (an increase of 450 vs. 81 kcal above baseline). Antonio et al.’s most recent investigation [92] was a 1-year crossover trial using resistance-trained subjects, comparing protein intakes of 3.3 vs. 2.5 g/kg. In agreement with previous findings, there were no differences in body composition (importantly, no significant increase in fat mass), despite a significantly higher caloric intake in HP vs. NP (an increase of 450 vs. 81 kcal above baseline). This study also addressed health concerns about long-term high protein intakes (3–4 times the RDA) by demonstrating no adverse effects on a comprehensive list of measured clinical markers, including a complete metabolic panel and blood lipid profile.

An in-patient, metabolic ward study by Bray et al. [76] compared 8 weeks of hypercaloric conditions with protein at 5 (LP), 15 (NP), and 25% of total energy (HP). All three groups gained total body weight, but LP lost 0.7 kg LM. Moreover, the NP and HP groups gained 2.87 and 3.98 kg LM, respectively. All three groups gained body fat (3.51 kg) with no significant difference between groups. These results are seemingly at odds with Antonio et al.’s observations [89–92]. However, aside from the tighter control and surveillance inherent with metabolic ward conditions, Bray et al.’s subjects were untrained and remained sedentary throughout the study. Antonio et al.’s well-trained subjects were undergoing intensive resistance training and could have had an advantage regarding fuel oxidation and preferential nutrient partitioning toward lean body mass.

Speculation over the fate of the extra protein consumed in the Antonio et al. studies [89–92] may include a higher thermic effect of feeding, increased non-exercise activity thermogenesis (NEAT), increased thermic effect of exercise (TEE), increased fecal energy excretion, reduced intake of the other macronutrients via increased satiety and suppressed hepatic lipogenesis. It should be noted as well that there might have been a misreporting of energy intake. Antonio et al.’s findings collectively suggest that the known thermic, satiating, and LM-preserving effects of dietary protein might be amplified in trained subjects undergoing progressive resistance exercise.

### Intermittent fasting

Intermittent fasting (IF) can be divided into three subclasses: alternate-day fasting (ADF), whole-day fasting (WDF), and time-restricted feeding (TRF) [93]. The most extensively studied IF variant is ADF, which typically involves a 24-hour fasting period alternated with a 24-hour feeding period. Complete compensatory intake on the feeding days (to offset the fasting days’ deficit) does not occur, and thus total weight loss and fat loss occurs on ADF. Lean mass retention has been a surprisingly positive effect of ADF [94–97]. However, lean mass loss in ADF conditions has also been observed by other investigators [98–100]. The latter effect might be attributable to more severe energy deficits. The more lean mass-friendly is an energy-restricted period (~25% of maintenance requirements, typically in the form of a single meal at lunchtime) alternated with a 24-hour ad libitum (as desired) feeding period. Recently, Catenacci et al. [97] reported that ADF with zero caloric intake on the fasting days alternated with ad libitum feeding days showed similar results to daily caloric restriction on body composition, and slightly outperformed daily caloric restriction after 6-months of unsupervised weight loss maintenance. On the note of alternating fasting and feeding periods of the same length, alternate-week energy restriction (1 week on ~1300 kcal/day, one week on the usual diet) has only a single study to date, but is worth mentioning since it was as effective as continuous energy restriction for reducing body weight and waist girth at 8 weeks and 1 year [101].

Whole-day fasting involves one to two 24-hour fasting periods throughout the week of otherwise maintenance intake to achieve an energy deficit. Of note, not all WDF studies involve zero energy intake during the ‘fasting’ days. Although WDF has been consistently effective for weight loss, Harvie et al. [102] saw no difference in body weight or body fat reduction between the WDF (2 ‘fasting’ days of ~647 kcal) group and controls when the weekly energy deficit was equated over a 6-month period. A subsequent study by Harvie et al. [103] compared daily energy restriction (DER) with two separate WDF diets: one with two structured energy-restricted ‘fasting’ days per week, and one whose 2 ‘fasting’ days consisted of ad libitum protein and unsaturated fat. Both WDF diets caused greater 3-month fat loss than DER (3.7 vs. 2.0 kg). An important detail here is that at 3 months, 70% of the fasting days were completed in the WDF groups while the DER group achieved their targeted caloric deficit only 39% of the trial.

Time-restricted feeding typically involves a fasting period of 16–20 hours and a feeding period of 4–8 hours daily. The most widely studied form of TRF is Ramadan fasting, which involves approximately 1 month of complete fasting (both food and fluid) from sunrise to sunset. Unsurprisingly, significant weight loss occurs, and this includes a reduction in lean mass as well as fat mass [104, 105]. Aside from Ramadan fasting studies, dedicated TRF research has been scarce until recently. An 8-week trial by Tinsley et al. [106] examined the effect of a 20-hour fasting/4-hour feeding protocol (20/4) done 4 days per week on recreationally active, but untrained subjects. No limitations were placed on the amounts and types of food consumed in the 4-hour eating window. A standardized resistance training program was administered 3 days per week. The TRF group lost body weight, due to a significantly lower energy intake (667 kcal less on fasting compared to non-fasting days). Cross sectional area of the biceps brachii and rectus femoris increased similarly in both the TRF and normal diet (ND) group. No significant changes in body composition (via DXA) were seen between groups. Despite a lack of statistical significance, there were notable effect size differences in lean soft tissue (ND gained 2.3 kg, while TRF lost 0.2 kg). Although both groups increased strength without significant between-group differences, effect sizes were greater in the TRF group for bench press endurance, hip sled endurance, and maximal hip sled strength. This finding should be viewed cautiously given the potential for greater and more variable neurological gains in untrained subjects.

A subsequent study by Moro et al. [107] found that in resistance-trained subjects on a standardized training protocol, a 16-hour fasting/8-hour feeding cycle (16/8) resulted in significantly greater FM loss in TRF vs. normal diet control group (ND) (1.62 vs. 0.31 kg), with no significant changes in LM in either group. TRF’s meals were consumed at 1 pm, 4 pm, and 8 pm. ND’s meals were consumed at 8 am, 1 pm, and 8 pm. Macronutrient intake between the TRF and ND groups was matched, unlike the aforementioned Tinsley et al. study [106] whereby protein intake was disparate and sub-optimal (1.0 g/kg in the TRF group and 1.4 g/kg in the ND control group). Subjects in the present study’s TRF and ND group consumed 1.93 and 1.89 g/kg, respectively. The mechanisms underlying these results are not clear. The authors speculated that increased adiponectin levels in the TRF group could have stimulated mitochondrial biogenesis via interacting with PPAR-gamma, in addition to adiponectin acting centrally to increase energy expenditure. However, the TRF group also experienced unfavorable changes such as decreased testosterone and triiodothyronine levels.

Seimon et al. [108] recently published the largest systematic review of IF research to date, comparing the effects of intermittent energy restriction (IER) to continuous energy restriction (CER) on body weight, body composition, and other clinical parameters. Their review included 40 studies in total, 12 of which directly compared an IER with a CER condition. They found that overall, the two diet types resulted in “apparently equivalent outcomes” in terms of body weight reduction and body composition change. Interestingly, IER was found to be superior at suppressing hunger. The authors speculated that this might be attributable to ketone production in the fasting phases. However, this effect was immaterial since on the whole, IF failed to result in superior improvements in body composition or greater weight loss compared to CER. Table 2 outlines the characteristics of the major diet archetypes.

**Table 2:** Diet categories

Diet	Composition	Strengths	Limitations
Low-energy diets (LED)	LED: 800–1200 kcal/day VLED: 400–800 kcal/day	Rapid weight loss (1.0–2.5 kg/week, diets involve premade products that eliminate or minimize the need for cooking and planning.	VLED have a higher risk for more severe side-effects, but do not necessary outperform LED in the long-term
Low-fat diets (LFD)	LFD: 25–30% fat VLFD: 10–20% fat	LFD have the support of the major health organizations due to their large evidence basis in the literature on health effects. Flexible macronutrient range. Does not indiscriminately vilify foods based on CHO content.	Upper limits of fat allowance may falsely convey the message that dietary fat is inherently antagonistic to body fat reduction. VLFD have a scarce evidence basis in terms of comparative effects on body composition, and extremes can challenge adherence.
Low-carbohydrate diets (LCD)	50–150 g CHO, or up to 40% of kcals from CHO	Defaults to higher protein intake. Large amount of flexibility in macronutrient proportion, and by extension, flexibility in food choices. Does not indiscriminately prohibit foods based on fat content.	Upper limits of CHO allowance may falsely convey the message that CHO is inherently antagonistic to body fat reduction.
Ketogenic diets (KD)	Maximum of ~50 g CHO Maximum of ~10% CHO	Defaults to higher protein intake. Suppresses appetite/controls hunger, causes spontaneous reductions in kcal intake under non-calorically restricted conditions. Simplifies the diet planning and decision-making process.	Excludes/minimizes high-CHO foods which can be nutrient dense and disease-preventive. Can compromise high-intensity training output. Has not shown superior effects on body composition compared to non-KD when protein and kcals are matched. Dietary extremes can challenge long-term adherence.
High-protein diets (HPD)	HPD: ≥ 25% of total kcals, or 1.2–1.6 g/kg (or more) Super HPD: > 3 g/kg	HPD have a substantial evidence basis for improving body composition compared to RDA levels (0.8 g/kg), especially when combined with training. Super-HPD have an emerging evidence basis for use in trained subjects seeking to maximize intake with minimal-to-positive impacts on body composition.	May cause spontaneous reductions in total energy intake that can antagonize the goal of weight gain. Potentially an economical challenge, depending on the sources. High protein intakes could potentially displace intake of other macronutrients, leading to sub-optimal intakes (especially CHO) for athletic performance goals.
Intermittent fasting (IF)	Alternate-day fasting (ADF): alternating 24-h fast, 24-h feed. Whole-day fasting (WDF): 1–2 complete days of fasting per week. Time-restricted feeding (TRF): 16–20-h fast, 4–8-h feed, daily.	ADF, WDF, and TRF have a relatively strong evidence basis for performing equally and sometimes outperforming daily caloric restriction for improving body composition. ADF and WDF have ad libitum feeding cycles and thus do not involve precise tracking of intake. TRF combined with training has an emerging evidence basis for the fat loss while maintaining strength.	Questions remain about whether IF could outperform daily linear or evenly distributed intakes for the goal of maximizing muscle strength and hypertrophy. IF warrants caution and careful planning in programs that require optimal athletic performance.

## Mechanisms governing changes in body composition vis a vis diet alterations

### Calories in/calories Out (CICO)

In its simplest form, CICO is an acronym for the idea that weight loss or gain is determined by a caloric deficit or surplus, regardless of diet composition. While this technically is true, it fails to account for the composition of the weight gained or lost, as well as the multitude of factors that drive eating behaviors that dictate caloric intake. Both voluntary and involuntary factors govern the “calories out” side of the equation, beginning with the varying metabolic cost of processing the macronutrients. As reported by Jéquier, the thermic effect of protein (expressed as a percentage of energy content) is 25–30%, carbohydrate is 6–8%, and fat is 2–3% [109]. However, Halton and Hu [110] reported greater variability, with the thermic effect of protein being 20–35%, carbohydrate at 5–15%, and fat being subject to debate since some investigators found a lower thermic effect than carbohydrate while others found no difference.

Variability in the thermic effect of fat can be attributed to differences in molecular structure that significantly alter its metabolism. For example, Seaton et al. [111] found that medium chain triglycerides (MCTs) produced a significantly greater thermic effect than long chain triglycerides during a 6-hour postprandial period (12 vs. 4% higher than basal oxygen consumption). Differences in the TEF of protein have also been observed in direct comparisons. Acheson et al. [112] demonstrated that within mixed-macronutrient meals (50% protein, 40% CHO, 10% fat) meals, whey had a higher thermic effect than casein, which had a higher thermic effect than soy protein. All protein sources had a higher thermic effect than an all-CHO meal. Importantly, the thermic effect of each macronutrient can vary within and across individuals [113]. In any case, protein has consistently shown a higher thermic effect than carbohydrate or fat. Alcohol has been reported to have a similar thermic effect to protein but with a wider range of 10–30% [114].

The thermic effect of food (TEF), also called diet-induced thermogenesis, is one of several components of EE. TEF represents approximately 8–15% of total daily energy expenditure (TDEE) [115]. The largest component of TDEE, at least among individuals not involved in extremely high volumes of exercise, is resting energy expenditure (REE), which is often mentioned interchangeably with resting metabolic rate (RMR) or basal metabolic rate (BMR). Basal metabolic rate is the energetic cost of the biological processes required for survival at rest. As a matter of technical trivia, BMR is measured in an overnight fasted state, lying supine at complete rest, in the postabsorptive state (the condition in which the gastrointestinal tract is empty of nutrients and body stores must supply required energy). REE/RMR represents fasted-state energy expenditure at rest at any time of the day, and can range 3–10% higher than BMR due to the residual influence of TEF and physical activity [116].

Basal metabolic rate typically amounts to 60–70% of TDEE. The other main component of TDEE is non-resting energy expenditure, which is comprised of 3 subcomponents: non-exercise activity thermogenesis (NEAT), exercise activity thermogenesis (EAT), and finally, TEF. NEAT encompasses the energy expenditure of occupation, leisure, basic activities of daily living, and unconscious/spontaneous activity such as fidgeting. While BMR and TEF are relatively static, NEAT and EAT vary widely within and across individuals. EAT has been reported to range from 15 to 30% of TDEE [115], but the role of NEAT is more easily overlooked. NEAT comprises ~15% of TDEE in sedentary individuals and perhaps 50% or more in highly active individuals [117]. The impact of NEAT can be substantial since it can vary by as much as 2000 kcals between individuals of similar size [118]. Table 3 outlines the components of TDEE, with examples of low, moderate, and high TDEE [115–117].

**Table 3:** Components of total daily energy expenditure

Component of TDEE	Percent of TDEE	Example: 1600 kcal TDEE	Example: 2600 kcal TDEE	Example: 3600 kcal TDEE
Thermic effect of food (TEF)	8–15	128–240	208–390	288–540
Exercise activity thermogenesis (EAT)	15–30	240–480	390–780	540–1080
Non-exercise activity thermogenesis (NEAT)	15–50	240–800	390–1300	540–1800
Basal metabolic rate (BMR)	60–70	960–1120	1560–1820	2160–2520

The oversimplification of the CICO concept has led to a call to “eat less, move more” as a solution to the obesity pandemic. While this advice technically *is* the answer, the challenge lies in programming the variables so that the desired energy balance is sustained over the long-term, and the targeted body composition is reached and maintained while preventing or minimizing REE losses. Involuntary adaptive shifts separate humans from machines. We differ from bomb calorimeters primarily due to our dynamic nature, which is based on the homeostatic drive toward survival. When hypocaloric conditions are imposed, energy expenditure has a tendency to decrease. Conversely, when a caloric surplus is imposed, EE has a tendency to increase. However, human energy balance has been called an asymmetric control system [119], because it tends to be lopsided in favor of more easily gaining weight but less easily losing weight. This asymmetry has been attributed to evolutionary pressures that selected the survival of “metabolically thrifty” individuals who more easily stored body fat during times of famine [120].

The degree of processing or refinement of foods can influence their thermic effect. Barr and Wright [121] found a diet-induced thermogenesis of 137 kcal for a ‘whole food’ meal, and 73 kcal for the processed food meal. The ‘whole food’ meal had 5% more protein, and 2.5 g more fiber, but these factors are too small to account for the substantial difference in postprandial energy expenditure. The authors speculated that the greater mechanized preparation of the processed food caused less peristalsis and a greater loss of bioactive compounds, resulting in fewer metabolites, thus requiring less enzyme activity. This would lead to more energetically efficient absorption and metabolism. It is important to note that this was not a comparison of a highly processed food versus a whole food. Both of the meals in the comparison were cheese sandwiches. One just happened to have less mechanical refinement, and slightly more fiber and protein. The results of this study imply that processed foods are more fattening or less effective for weight management. However, the contrary has been demonstrated. Meal replacement products (powders, shakes, and bars) have matched or outperformed the effectiveness of whole food-based diets for weight loss and weight loss maintenance [82, 122, 123].

An awareness of tissue-specific metabolism can be helpful in understanding the resting metabolic benefits of improving body composition. It can also serve to clarify the widely misunderstood and often overestimated contribution of muscle to REE. McClave and Snider [124] reported that the greatest contributors to REE, per unit of mass, are the heart and kidneys, each spending approximately 400 kcal/kg/day. Next in the hierarchy are the brain and the liver, at 240 and 200 kcal/kg/day, respectively. These four organs constitute up to 70–80% of REE. In contrast, muscle and adipose tissue expend 13 and 4.5 kcal/kg/day, respectively. This should debunk the notion that increases in muscle mass give individuals the license to reduce dietary discretion. Even a relatively significant muscular gain of 5 kg would increase REE by only ~65 kcal/day. However, on a *net* basis (accounting for the total mass of each tissue in the body), muscle, brain, and liver are the top-3 contributors to overall REE. Thus, substantial losses in LM – including muscle – can meaningfully impact REE. Finally, it should be noted that tissue-specific EE can vary according to obese vs. non-obese status, advanced age, and to a lesser degree, sex [125]. Table 4 outlines the contribution of organs and tissues to REE in healthy adult humans [124].

**Table 4:** Energy Expenditure of Different Tissues/Organs

Organ or tissue	Metabolic rate (kcal/kg/day)	% Overall REE	Weight (kg)	% of Total body weight
Adipose	4.5	4	15	21.4
Other (bone, skin, intestine, glands)	12	16	23.2	33.1
Muscle	13	22	28	40.0
Liver	200	21	1.8	2.6
Brain	240	22	1.4	2.0
Heart	400	9	0.3	0.5
Kidneys	400	8	0.3	0.5

### Adaptations to underfeeding

Humans have a remarkable ability to maintain a relatively constant body weight through adult life despite wide variations in daily energy intake and expenditure. This indicates a highly sophisticated integration of systems that tirelessly auto-regulate homeostasis. In the case of hypocaloric conditions, the body up-regulates hunger and down-regulates energy expenditure. The integration of physiological factors regulating the body’s defense against weight loss (and also weight gain) is symphonic. The central nervous system ‘communicates’ with the adipose tissue, gastrointestinal tract and other organs in an effort to defend against homeostatic changes. This regulatory system is influenced by nutritional, behavioral, autonomic, and endocrine factors [126].

The changes in EE are not always completely accounted for by changes in lean mass and fat mass. Therefore, in the context of hypocaloric diets, *adaptive thermogenesis* (AT) is a term used to describe the gray area where losses in metabolic tissue cannot simply explain reduced EE. In lean and obese subjects, maintaining a drop of ≥10% of total body weight results in a ~20–25% decrease in TDEE [127]. AT is a 10–15% drop in TDEE beyond what is predicted by losses in LM and FM as a result of maintaining a loss of ≥10% of total body weight. In weight-reduced subjects, the vast majority of (85–90%) of AT is due to decreased non-resting energy expenditure. The mechanisms underlying AT are unclear, but speculations include increased sympathetic drive and decreased thyroid activity. A classic experiment by Leibel et al. [128] demonstrated that in obese subjects, a 10% or greater weight loss resulted in a 15% greater EE reduction than predicted by body composition change. However, these subjects were put on an 800 kcal liquid diet composed of 15% protein, 45% CHO, and 40% fat. Imposed reductions in EE via low-protein VLED do not necessarily reflect what is possible under conditions involving better macronutrient targets and proper training.

In contrast to Leibel et al.’s findings [128] and a recent study by Rosenbaum and Leibel [129] using the same low-protein VLED, Bryner et al. [25] observed an *increased* RMR by the end of 12 weeks in subjects on an 800 liquid kcal diet. The discrepancy between Bryner et al.’s results and those of Leibel et al. can be explained by better macronutrient distribution and the implementation of resistance exercise. Bryner et al.’s VLED was composed of 40% protein, while Leibel et al.’s was 15% (30 g protein). Bryner’s subjects underwent full-body resistance training three times per week, while Leibel’s design neglected exercise programming altogether.

More recently, Camps et al. [130] found that after significant weight loss resulting from 8 weeks on a VLED, reduced EE beyond what was predicted was still present after a year. While this can be viewed as the unfortunate persistence of weight loss-induced AT, the actual difference in RMR at baseline versus 52 weeks was a reduction of 81 kcal, where total weight loss was 5.4 kg (5.0 kg of which was FM). However, it is worth reiterating that higher protein alongside resistance training has been shown to prevent this impairment despite severe caloric restriction [25]. As it stands, the subjects were not engaged in structured exercise at any point (let alone a resistance training program that would support the metabolic activity of lean mass), and the details of their maintenance diet were not reported. In all likelihood, it was not optimized in terms of macronutrition.

Misreporting energy intake and output is a common occurrence that has the potential to be mistaken for metabolic adaptation. For example, Lichtman et al. [131] used indirect calorimetry and doubly labeled water to objectively assess energy intake and output in obese subjects with a history of diet resistance, and a claimed intake of less than 1200 kcal/day. In the experimental group, no subject had a TEE more than 9.6% below the predicted values (average TEE was 2468 kcal), and no subject had a RMR more than 10.4% below predicted values. It was determined that instead of some defect in thermogenesis, subjects under-reported their intake by an average of 47% (1053 kcal/day), and over-reported physical activity by 51% (251 kcal/day). Clearly, the gap between perceived compliance and actual compliance remains a major challenge to the goal of improving body composition.

### Adaptations to overfeeding

In hypocaloric conditions, adaptive thermogenesis (AT) is a misnomer; it would more accurately be called *adaptive thermoreduction* due to a reduction in energy expenditure in response to reductions in energy intake. However, “adaptive thermogenesis” would be a more appropriate term for describing the production of heat in response to reductions in environmental temperature, or hypercaloric conditions. Joosen and Westerterp [132] examined the literature (11 studies) to see if AT existed in overfeeding experiments. No evidence beyond the theoretical costs of increased body size and TEF were found. Nevertheless, there is substantial interindividual variability in the energetic response to overfeeding. Some individuals appear to be resistant to weight/fat gain, showing a concurrent increase in expenditure alongside increased intake. Others show less homeostatic drive and greater efficiency of energy storage. This interindividual variability is due, at least in part, to differences in NEAT.

A question relevant to fitness, sports nutrition, and body composition-oriented goals is whether so-called “hardgainers” have a metabolic impediment against weight gain or whether this is a lack of conscious discipline to sustain a caloric surplus. It is possible that conscious and unconscious increases in NEAT can pose a significant challenge to weight gain. A prime illustration of this is a study by Levine et al. [133], who fed non-obese adults 1000 kcal above their maintenance needs for eight weeks. On average, 432 kcal were stored, and 531 kcal were burned. Nearly two-thirds of the latter (336 kcal) was attributable to NEAT, which on the upper end of the range was 692 kcal/day. This finding explains why some individuals can purposely increase daily caloric intake and still experience a lack of weight gain. Unbeknownst to them, increased NEAT can negate the targeted caloric surplus.

The partitioning of a chronic energy surplus into the various tissue compartments is an important yet understudied area. Rosqvistet al. [134] compared the effects of hypercaloric diets fortified with polyunsaturated fatty acid (PUFA) versus saturated fatty acid (SFA). Despite similar gains in total body weight (1.6 kg, via an additional 750 kcal/day from fat-fortified muffins), the ratio of LM:FM gained in the PUFA group was 1:1, whereas it was 1:4 in the SFA group, indicating a better LM-partitioning effect of surplus energy from PUFA. Furthermore, liver fat and visceral fat deposition were significantly greater in SFA. The authors speculated that a greater oxidation of PUFA might have decreased the production of non-esterified fatty acids, which in turn could have lowered hepatic triacylglycerol synthesis. Caution is warranted when attempting to generalize these results beyond the fat sources used (palm oil for SFA, sunflower oil for PUFA).

Chronic overfeeding adaptations can also vary according to training status. Garthe et al. [135] compared the 12-week effects of 3585 kcal/day (544 kcal increase from baseline intake) in a nutritionally counseled group vs. 2964 kcal/day (128 kcal decrease from baseline) in the ad libitum group, without counseling. Elite athletes in a variety of sports were used. Lean mass gains were slightly but not significantly higher in the nutritionally counseled group (1.7 kg vs. 1.2 kg), but fat gain was also significantly higher (1.1 kg vs. 0.2 kg). In contrast, Rozenek et al. [136] compared the 8-week effects of a massive caloric surplus (2010 kcal/day) consisting of 356 g carbohydrate, 106 g protein, and 18 g fat (CHO-PRO), or an isocaloric higher-carb treatment (CHO) consisting of 450 g carbohydrate, 24 g protein, and 14 g fat. A non-supplemented control group was included in the comparison, and this group underwent the same progressive resistance training protocol as the treatment groups. In contrast to Garthe et al.’s findings [135], Roznek et al.’s subjects gained almost exclusively LM in the CHO-PRO group (2.9 kg) with very little fat mass gain (0.2 kg). The CHO group showed slightly better results than CHO-PRO, although not to a statistically significant degree (3.4 kg LM gain, 0.3 kg FM loss). It was speculated that both groups consumed adequate protein at baseline (1.6 g/kg), so the additional protein in CHO-PRO (which increased protein intake to 2.9 g/kg) did not further enhance LM gains. Garthe et al. [135] saw a significant amount of fat gain alongside the lean gain despite a much smaller caloric surplus (544 vs. 2010 kcal above maintenance). However, Garthe et al.’s subjects were elite athletes, while Rozenek et al.’s subjects were untrained, so it is possible that they were better primed for more dramatic progress in both departments (LM gain with minimal FM gain) despite the massive caloric surplus.

It can be argued that sustaining a caloric surplus is not necessary for muscle anabolism since LM gains have been reported in the literature during hypocaloric conditions [26, 80, 137, 138]. However, Pasiakos et al. [139] demonstrated a significant decrease in muscle protein synthesis and lower phosphorylation of associated intracellular signaling proteins during 10 days of a moderate energy deficit (80% of estimated energy requirements). Therefore, it is likely that diets seeking to optimize rates of LM gain are compromised by sustained caloric deficits, and optimized by sustained caloric surpluses to facilitate anabolic processes and support increasing training demands.

## Summary and conclusions

### Summary

Understanding how various diet types affect body composition is of utmost importance to researchers and practitioners. Ultimately, the interpretation of the data and implementation of the procedures determine the progress made by clients, patients, and the public. Fortunately, the current body of research is rich with information that can guide evidence-based theory and practice. Body composition assessment methods vary in their level of precision, reliability, and availability. Each method has its strengths and weaknesses. No single approach is ideal for all circumstances. Rather, the practitioner or researcher must employ the most practical option for the assessment needs of the individuals at hand, in order to achieve consistency in the face of inherent limitations and logistical considerations such as financial expense and technician skill. The various diet archetypes are wide-ranging in total energy and macronutrient distribution. Each type carries varying degrees of supporting data, and varying degrees of unfounded claims. Common threads run through the diets in terms of mechanism of action for weight loss and weight gain (i.e., sustained hypocaloric versus hypercaloric conditions), but there are also potentially unique means by which certain diets achieve their intended objectives (e.g., factors that facilitate greater satiety, ease of compliance, support of training demands, etc.).

### Conclusions and recommendations

- There is a vast multitude of diets. In addition, there are numerous subtypes that fall under the major diet archetypes. Practitioners, clinicians, and researchers need to maintain a grasp of the claims versus the evidence underlying each archetype to properly guide science-based practical and educational objectives with clients, patients, and the public.
- All body composition assessment methods have strengths and limitations. Thus, the selection of the method should weigh practicality and consistency with the prohibitive potential of cost, invasiveness, availability, reproducibility, and technician skill requirements. Ultimately, the needs of the client, patient, or research question should be matched with the chosen method; individualization and environmental considerations are essential.
- Diets focused primarily on FM loss (and weight loss beyond initial reductions in body water) operate under the fundamental mechanism of a sustained caloric deficit. This net hypocaloric balance can either be imposed linearly/daily, or non-linearly over the course of the week. The higher the baseline FM level, the more aggressively the caloric deficit may be imposed [27]. As subjects get leaner, slower rates of weight loss can better preserve LM, as in Garthe et al.’s example of a weekly reduction of 0.7% of body weight outperforming 1.4% [138]. Helms et al. [140] similarly suggested a weekly rate of 0.5–1.0% of body weight for bodybuilders in contest preparation.
- Although LM gains have been reported in the literature during hypocaloric conditions, diets primarily focused on LM gain are likely optimized via sustained caloric surplus to facilitate anabolic processes and support increasing training demands. The composition and magnitude of the surplus, the inclusion of an exercise program, as well as training status of the subjects can influence the nature of the gains. Larger caloric surpluses are more appropriate for untrained subjects who are primed for more dramatic progress in LM gain [136] and for those with a high level of NEAT [133]. On the other hand, smaller caloric surpluses are appropriate for more advanced trainees who may be at a higher risk for undue FM gain during aggressive hypercaloric conditions [135]. It should be noted that not all trainees will fit within this general framework. Some novices might require smaller surpluses while some advanced trainees will require larger surpluses in order to push muscular gains forward. It is the job of the practitioner to tailor programs to the inevitable variability of individual response.
- A wide range of dietary approaches (low-fat to low-carbohydrate/ketogenic, and all points between) can be similarly effective for improving body composition, and this allows flexibility with program design. To date, no controlled, inpatient isocaloric diet comparison where protein is matched between groups has reported a clinically meaningful fat loss or thermic advantage to the lower-carbohydrate or ketogenic diet [60]. The collective evidence in this vein invalidates the carbohydrate-insulin hypothesis of obesity. However, ketogenic diets have shown appetite-suppressing potential exemplified by spontaneous caloric intake reductions in subjects on ketogenic diets without purposeful caloric restriction. Athletic performance is a separate goal with varying demands on carbohydrate availability depending on the nature of the sport. Carbohydrate restriction can have an ergolytic potential, particularly for endurance sports. Effects of carbohydrate restriction on strength and power warrant further research.
- Increasing dietary protein to levels significantly beyond current recommendations for athletic populations may improve body composition. The ISSN’s original 2007 position stand on protein intake (1.4–2.0 g/kg) [141] has gained further support from subsequent investigations arriving at similar requirements in athletic populations [88, 140, 142–145]. Higher protein intakes (2.3–3.1 g/kg FFM) may be required to maximize muscle retention in lean, resistance-trained subjects in hypocaloric conditions [88]. Emerging research on very high protein intakes (>3 g/kg) has demonstrated that the known thermic, satiating, and LM-preserving effects of dietary protein might be amplified in resistance-training subjects. It is possible that protein-targeted caloric surpluses in outpatient settings have resulted in eucaloric balance via satiety-mediated decreases in total calories, increased heat dissipation, and/or LM gain with concurrent FM loss [89, 90, 92].
- Time-restricted feeding (a variant of IF) combined with resistance training is an emerging area of research that has thus far shown mixed results [106, 107]. However, the body of intermittent caloric restriction research, on the whole, has indicated no significant advantage over daily caloric restriction for improving body composition [108]. Therefore, programming of linear versus nonlinear caloric deficits should be determined by individual preference, tolerance, and athletic goals. Adequate protein, resistance training, and an appropriate rate of weight loss should be the primary focus for achieving the objective of LM retention (or gain) during FM loss.
- The long-term success of the diet depends upon how effectively the mitigating factors of homeostatic drive are suppressed or circumvented. Hypocaloric conditions for fat loss have resulted in adaptive thermogenesis – a larger than predicted decrease in energy expenditure (10–15% below the predicted drop in TDEE after accounting for LM and FM loss). However, the majority of the existing research showing AT has involved diets that combine aggressive caloric restriction with low protein intakes and an absence of resistance training; therefore, essentially creating a perfect storm for the slowing of metabolism. Research that has mindfully included resistance training and adequate protein has circumvented the problem of AT [25] and LM loss [26], despite very low-calorie intakes.

### Perspectives and future directions

It is important to maintain the proper “big picture” perspective of the various programming elements to productively direct the right amount of focus and effort. When ranking nutritional factors by importance or impact on body composition, a cake analogy is simple, vivid, and memorable. The cake is total daily macronutrition (and micronutrition), the icing is the specific timing and distribution of nutrient intake through the day, and the sprinkles are supplements that might help trainees clinch the competitive edge. An ideal yet not always feasible scenario is a multidisciplinary team approach to client or patient care (i.e., dietitian, personal trainer, psychologist, physician). This makes the most efficient use of expertise in covering the various facets of lifestyle modification, and when necessary, medical intervention [146].

Research on dietary effects on body composition has plenty of gray areas and unbeaten paths ripe for investigation. There is still a general lack of research on women and older populations. Studies on the effect of different within-day meal frequencies and nutrient distributions in varying energetic balances combined with resistance or endurance training are still rather scarce. Linear versus nonlinear macronutrient intakes through the week, combined with exercise, is still an untapped area in research despite being widely practiced in the real-world. Therefore, while a certain amount of our current knowledge will remain static, scientists both in the lab and in the field should stay vigilant and open-minded to the modification and falsification of models and beliefs as the march of research continues.

## Abbreviations

2C: Two-compartment model
3C: Three-compartment model
4C: Four-compartment model
AMDR: Acceptable Macronutrient Distribution Ranges
AT: Adaptive thermogenesis
BIA: Bioelectrical impedance analysis
BIS: Bioimpedance spectroscopy
BMR: Basal metabolic rate
CHO: Carbohydrate
CICO: Calories-in/calories-out
EAT: Exercise activity thermogenesis
EE: Energy expenditure
FFM: Fat-free mass, used interchangeably with lean mass (LM) according to how it was reported in the literature
FM: Fat mass
HP: High-protein
IER: Intermittent energy restriction
IF: Intermittent fasting
KD: Ketogenic diet
LCD: Low-carbohydrate diet
LM: Lean mass
LP: Low-protein
NEAT: Non-exercise activity thermogenesis
PUFA: Polyunsaturated fatty acid
RDA: Recommended dietary allowance
REE: Resting energy expenditure
RMR: Resting metabolic rate
SFA: Saturated fatty acid
SM: Skeletal muscle
TBW: Total body water
TDEE: Total daily energy expenditure
TEE: Thermic effect of exercise
TEF: Thermic effect of food
VLED: Very-low-energy diet
